# Navigating the Chemical Space and Chemical Multiverse of a Unified Latin American Natural Product Database: LANaPDB

**DOI:** 10.3390/ph16101388

**Published:** 2023-09-30

**Authors:** Alejandro Gómez-García, Daniel A. Acuña Jiménez, William J. Zamora, Haruna L. Barazorda-Ccahuana, Miguel Á. Chávez-Fumagalli, Marilia Valli, Adriano D. Andricopulo, Vanderlan da S. Bolzani, Dionisio A. Olmedo, Pablo N. Solís, Marvin J. Núñez, Johny R. Rodríguez Pérez, Hoover A. Valencia Sánchez, Héctor F. Cortés Hernández, José L. Medina-Franco

**Affiliations:** 1 DIFACQUIM Research Group, Department of Pharmacy, School of Chemistry, Universidad Nacional Autónoma de México Avenida Universidad 3000, Mexico City 04510, Mexico; alex.go.ga21@hotmail.com; 2 CBio3 Laboratory, School of Chemistry, University of Costa Rica, San Pedro, San José 11501-2060, Costa Rica; daniel.acunajimenez@ucr.ac.cr (D.A.A.J.); william.zamoraramirez@ucr.ac.cr (W.J.Z.); 3 Laboratory of Computational Toxicology and Artificial Intelligence (LaToxCIA), Biological Testing Laboratory (LEBi), University of Costa Rica, San Pedro, San José 11501-2060, Costa Rica; 4 Advanced Computing Lab (CNCA), National High Technology Center (CeNAT), Pavas, San José 1174-1200, Costa Rica; 5 Computational Biology and Chemistry Research Group, Vicerrectorado de Investigación, Universidad Católica de Santa Maria, Arequipa 04000, Peru; hbarazorda@ucsm.edu.pe (H.L.B.-C.); mchavezf@ucsm.edu.pe (M.Á.C.-F.); 6 Laboratory of Medicinal and Computational Chemistry (LQMC), Centre for Research and Innovation in Biodiversity and Drug Discovery (CIBFar), São Carlos Institute of Physics (IFSC), University of São Paulo (USP), Av. João Dagnone, 1100, São Carlos 13563-120, SP, Brazil; marilia.valli@ifsc.usp.br (M.V.); aandrico@ifsc.usp.br (A.D.A.); 7 Nuclei of Bioassays, Biosynthesis and Ecophysiology of Natural Products (NuBBE), Department of Organic Chemistry, Institute of Chemistry, São Paulo State University (UNESP), Av. Prof. Francisco Degni, 55, Araraquara 14800-900, SP, Brazil; vanderlan.bolzani@unesp.br; 8 Center for Pharmacognostic Research on Panamanian Flora (CIFLORPAN), College of Pharmacy, University of Panama, Av. Manuel E. Batista and Jose De Fabrega, Panama City 3366, Panama; dionisio.olmedo@up.ac.pa (D.A.O.); pablonsolis@gmail.com (P.N.S.); 9 Natural Product Research Laboratory, School of Chemistry and Pharmacy, University of El Salvador, Final Ave. Mártires Estudiantes del 30 de Julio, San Salvador 01101, El Salvador; marvin.nunez@ues.edu.sv; 10 GIFES Research Group, School of Chemistry Technology, Universidad Tecnológica de Pereira, Pereira 660003, Colombia; johny.rodriguez@utp.edu.co (J.R.R.P.); hvalencia@utp.edu.co (H.A.V.S.); hfcortes@utp.edu.co (H.F.C.H.); 11 GIEPRONAL Research Group, School of Basic Sciences, Technology and Engineering, Universidad Nacional Abierta y a Distancia, Dosquebradas 661001, Colombia

**Keywords:** chemical multiverse, chemical space, chemoinformatics, databases, diversity, drug discovery, Latin America, natural products, virtual screening

## Abstract

The number of databases of natural products (NPs) has increased substantially. Latin America is extraordinarily rich in biodiversity, enabling the identification of novel NPs, which has encouraged both the development of databases and the implementation of those that are being created or are under development. In a collective effort from several Latin American countries, herein we introduce the first version of the Latin American Natural Products Database (LANaPDB), a public compound collection that gathers the chemical information of NPs contained in diverse databases from this geographical region. The current version of LANaPDB unifies the information from six countries and contains 12,959 chemical structures. The structural classification showed that the most abundant compounds are the terpenoids (63.2%), phenylpropanoids (18%) and alkaloids (11.8%). From the analysis of the distribution of properties of pharmaceutical interest, it was observed that many LANaPDB compounds satisfy some drug-like rules of thumb for physicochemical properties. The concept of the chemical multiverse was employed to generate multiple chemical spaces from two different fingerprints and two dimensionality reduction techniques. Comparing LANaPDB with FDA-approved drugs and the major open-access repository of NPs, COCONUT, it was concluded that the chemical space covered by LANaPDB completely overlaps with COCONUT and, in some regions, with FDA-approved drugs. LANaPDB will be updated, adding more compounds from each database, plus the addition of databases from other Latin American countries.

## 1. Introduction

Historically, natural products (NPs) have been the biggest source of bioactive compounds for medicinal chemistry. For instance, in cancer research, in the lapse of time from 1946 to 1980, seventy-five small molecules were approved worldwide, of which 53% were unaltered NPs or natural product (NP) derivatives. Moreover, from 1981 to 2019, of the 185 small molecules approved to treat cancer, 64.9% were unaltered NPs and synthetic drugs with a NP pharmacophore [1]. Another example is the actual development of new promising antibiotics against drug-resistant bacteria from NPs [2]. Furthermore, in a recent review, it was shown that 697 natural steroidal alkaloids were isolated and characterized with various biological activities, from 1926 to 2021 [3]. The bioactive compounds encompass marine [4,5], fungal [6,7], bacteria [8], plants [9] and endogenous substances produced by human and animal sources [10], including venoms and poisons produced by different animals [11]. Even, as recently reviewed, the fruit peels are a source of bioactive compounds, which, in many instances, display better biological and pharmacological applications than the compounds of other sections of the fruit [12].

There are several approaches to the drug discovery process for NPs. The information of the therapeutic effects or even the side effects can serve as a starting point [10]. The stress-driven growth of plants and microorganisms stimulates the production of secondary metabolites with biological activities different to the primary metabolites produced under normal conditions [13]. Drug repositioning of NPs is another option, which offers lower development costs and shorter time frames [14]. Moreover, NPs are a rich source of “privileged scaffolds”, structures capable of providing useful ligands for more than one receptor [15]. Privileged scaffolds are useful because they can serve as a core structure to construct compound libraries around them [1]. Some examples of privileged scaffolds that are actually used for drug design purposes are the alkaloid, terpenoid polyketide and phenylpropanoid structures [16]. Furthermore, the preparation of biologically relevant small-molecule libraries through unprecedented combinations of NP fragments to afford novel scaffolds that do not occur in nature is an approach that involves the preparation of molecules named “pseudo-natural products” (pseudo-NPs) [17]. The pseudo-NPs retain the biological relevance of NPs, yet exhibit structures and bioactivities not accessible to nature or through the use of existing design strategies. Pseudo-NPs may display unexpected bioactivities that differ from the activities of the NPs from which their fragments are derived. That is why their bioactivity should be monitored in a wide biological space through different biochemical and biological assays. Most of the pseudo-NP collections fall within the “Lipinski rule of 5” (Ro5) space, showing advantageous physicochemical “drug-like” properties. For the design of pseudo-NP libraries, it is important to consider that the combination of biosynthetically unrelated NP fragments may be beneficial for novel bioactivity, maximizing the biological relevance of the resulting pseudo-NP scaffold. Chromopynones, indotropanes, pyrrotropanes and pyrroquinolinones are part of pseudo-NP collections that have been developed for the first time, an unprecedented combination of these scaffolds, resulting in totally new chemical entities [18,19]. 

Over time, NPs have been a source of compounds with therapeutical effects and many of them, later, end up converting into approved drugs. Some of them have been approved as drugs without suffering a structural modification. In other cases, they serve as starting points that, later, with further structural modifications, are approved as drugs. Sometimes, bioactive molecules from NPs lack suitable physicochemical properties, and their synthetic complexity may hinder their direct use as therapeutics. In this case, to be developed as drug candidates, NPs need to go through an optimization process that usually involves structural modifications to improve one or more of the following characteristics: potency, selectivity, solubility, metabolic and chemical stability; and the removal (or at least significant reduction) of toxicity [20]. This is usually done by decreasing the molecular size, eliminating the unnecessary functional groups and chiral centers and introducing nitrogen atoms if they are needed, because in the NPs the presence of nitrogen is limited.

To date, the discovery process of more than seventy commercialized drugs has included the rational use of at least a computational method [21]. Computer-aided drug design (CADD) has the potential to reduce the cost and decrease the time required for the drug design process, e.g., the hit identification rate for high-throughput screening (HTS) to discover novel inhibitors for the enzyme protein tyrosine phosphatase-1B is only 0.021% and the one for molecular docking is 34.8% [22]. Some crucial resources in CADD are the databases of chemical compounds, including NP databases. From the compound databases, it is possible to identify potential hit molecules through several virtual screening (VS) techniques [23,24], including the training of artificial intelligence (AI) algorithms [25]. When the compound databases are annotated with biological activity (or other property of relevance), it is possible to use the data to measure structure–activity (property) relationships and develop predictive models. 

VS techniques are usually classified into two major categories: structure-based (SBVS) and ligand-based (LBVS). In general, SBVS is more suitable for finding structurally novel ligands, and is the preferred method when the three-dimensional (3D) structure of the target protein has been characterized experimentally [23]. When the structure of the target is unknown, or its prediction by structure-based methods is challenging, LBVS is the choice [24]. LBVS is based on the assumption that molecules with similar structures exhibit similar behavior. Among the LBVS techniques are the quantitative structure–activity relationship (QSAR) [23] and quantitative structure–property relationship (QSRP) [26] studies. QSAR/QSPR studies aim to find a mathematical association between the molecule structure and a given property, such as biological activity [24]. In this sense, the bioactivity and chemical information (i.e., chemogenomic) databases are crucial to allow the creation of QSAR/QSPR models that predict certain pharmacological activity or properties of pharmaceutical interest for a determined molecule or set of analog molecules. 

Another important application of the databases in the drug discovery process is the training of AI algorithms. AI encompasses a set of computational algorithms that allow computers to simulate human cognitive abilities, such as learning from experience and solving problems [27]. Among the LBVS is the AI-based QSAR, the creation and training of these models relies on the data found in the bioactivity databases. AI can be applied to the SBVS; specifically, to the docking of the protein-ligand complexes [25]. AI-based scoring functions have shown better performance in benchmark studies [28,29]. The creation of AI-based scoring functions depends on the availability of the required data in the database to train the model. AI algorithms have already been applied in the drug discovery process from NPs. To name a few: data-mining into traditional medicines and peer-reviewed articles, prediction of chemical structures from microbial genomes, automation of NPs dereplication process, encoding NPs into molecular representations, vectorization of NPs with molecular descriptors, mapping of NPs in the chemical space, engineering likeness scores, deorphanization and de novo generation of natural product-inspired compounds [30]. The report on using AI to create models that allow for the prediction of biological effects from NPs has been rising in recent years. The application of AI models to predict biological effects of molecules, toxicity, drug–target and drug–drug interactions has been reviewed [31]. 

From 2003 to 2018, 104 research articles reported the identification of potential drug candidates from NP databases by using computational tools [32]. Moreover, during the current pandemic outbreak, NPs have been a rich source for discovering potential lead compounds against severe acute respiratory syndrome coronavirus 2 (SARS-CoV-2) [33,34,35,36]. Nonetheless, the computational methods, such as the VS techniques, are valuable tools and should be implemented together with the in vivo or in vitro assays to increase the success rate in the identification of bioactive molecules. Recently (August 2022), the outperformance of combining the computational methods with the biological assays was shown [37]. There are still many challenges in the identification of bioactive compounds from the screening of large collections of compounds. In the HTS approach, a common problem is the presence of frequent hitters: compounds that would form aggregates, react with proteins or interfere in screening assays, leading to false positives [38]. Combining the HTS approach with computational methods can lead to the identification and elimination of the frequent hitters prior to the start of the HTS study.

Between 2000 and 2019, one hundred and twenty-three commercial and public NP databases have been published. Among them, ninety-eight are still somehow accessible (online or under request access), ninety-two are free access and only fifty contain molecular structures that can be retrieved for a chemoinformatic analysis [39]. Examples of the most representative open-access NP databases include: The Collection of Open Natural Products (COCONUT) [40], which is a major repository containing more than 411,000 NPs collected from 50 open access NP databases. The Universal Natural Product Database [41] is a compilation that tries to gather all the known NPs—it has more than 229,000 NPs. It is not yet accessible through the link in the original publication, nevertheless, it is contained and maintained on the ISDB website [42]. The SuperNatural 3.0 [43] database contains over 449,048 NPs—it provides a bulk download and has information on pathways, mechanism of action, toxicity and vendor information if available. The ZINC [44] database has over 80,000 NPs, approximately 48,000 purchasable. Moreover, it contains some NP databases that are no longer accessible through the link provided in the original publication, e.g., the Herbal Ingredient Targets [45] and the Herbal Ingredients In Vivo Metabolism database [46], which mostly contain NPs from Chinese plants. A practical application of the public molecular databases that exemplifies their utility in the drug discovery area is the recent identification of inhibitors of the human immunodeficiency virus-1 from 1.6 million commercially available drug-like compounds from the ZINC database. Moreover, there are NP databases that contain compounds isolated and characterized in certain geographical areas. That is the case of China, where multiple compound databases containing only NPs from this country have been published [47,48,49,50,51,52,53], of which, TCM@Taiwan [54] is the largest, containing 58,000 compounds. There are two databases of NPs from India, IMPPAT [55], composed of approximately 10,000 phytochemicals extracted from 1700 medicinal plants, and MedPServer [56], containing 1124 NPs. Regarding NPs from Africa, there are several NP databases [57,58,59,60,61,62], of which, AfroDB [63] is the most extensive, containing over one thousand NPs. Recently, Phyto4Health was published [64], an NP database with 3128 NPs isolated from medicinal plants of Russia.

**Figure 1:**
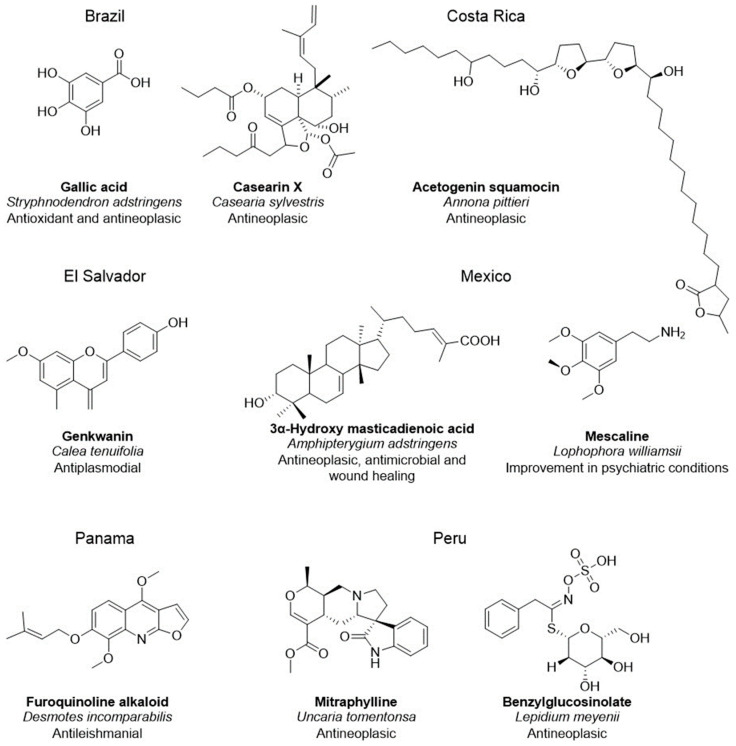
Active compounds of representative medicinal plants from Latin American countries and some of their therapeutic effects described in the literature. Brazil (gallic acid [91] and casearin x [92]), Costa Rica (acetogenin squamocin [93]), El Salvador (Genkwanin [94]), Mexico (3α-hydroxy masticadienoic acid [95] and mescaline [96]), Panama (furoquinoline alkaloid [97]) and Peru (mitraphylline [98] and benzylglucosinolate [99]).

Latin America contains at least a third of the global biodiversity [65]; in fact, half of the countries have been classified as megadiverse: Bolivia, Brazil, Colombia, Costa Rica, Ecuador, Mexico, Peru and Venezuela) [66]. Therefore, Latin America represents a large source of bioactive molecules and potential drug candidates (Figure 1). There have been published databases containing NPs from some Latin American countries, such as NaturAr [67] (Argentina), NuBBE_DB_ [68,69], SistematX [70,71], UEFS [72] (Brazil), CIFPMA [73,74] (Panama), PeruNPDB [75], (Peru), UNIIQUIM [76] and BIOFACQUIM [77,78] (Mexico). Recently, the present state of the art in developing Latin American NP databases and their practical applications to the drug discovery area were reviewed [79]. Multiple drug candidates have been identified from the Latin American NP databases as therapeutic agents for diseases caused by infectious agents (Chagas disease [80,81], tuberculosis [82], Leishmaniasis [83,84], schistosomiasis [85], coronavirus disease [86], human immunodeficiency virus infection and acquired immunodeficiency syndrome, hepatitis B and C) [87], pain [88], obesity, diabetes, hyperlipoproteinemia, cancer and age-related diseases [89,90]. 

The long-term goal of the project is to collect, unify and standardize the Latin American NP collections available in the public domain into one public database. In this study, we report significant advances towards this goal through the assembly of the first version of the unified database, herein called the Latin American Natural Products Database (LANaPD). We report its curation, standardization and a comprehensive analysis of nine compound databases, totaling 12,959 unique molecules. As part of this study, we analyzed the structural content and determined some physicochemical properties of pharmaceutical interest of the compounds in LANaPDB. We also represent coverage in the chemical space of compounds in LANaPDB using the concept of a chemical multiverse [100]. The database is freely available at https://github.com/alexgoga21/LaNaPDB (accessed on 28 September 2023).

## 2. Results and Discussion

### 2.1. Bioactive Compounds from Latin American Natural Product Databases

Bioactive compounds have been identified from Latin American NP databases. Ten compounds against *Trypanosoma cruzi* [80] have been identified from the NuBBE_DB_ database. Moreover, 13 compounds against *Mycobacterium tuberculosis* were identified in another study from NuBBE_DB_ [82]. 

Several bioactive compounds have been identified from five VS studies from the SistematX database. The bioactive compounds found include 1306 sesquiterpene lactones with potential activity against Trypanosoma cruzi [81]. In another VS study, 13 promising antileishmanial compounds were identified [83]. In the third VS study, the researchers looked for compounds against Schistosoma mansoni; from this, five compounds were identified with potential schistosomicidal activity [85]. In the fourth VS study, 19 compounds were identified as potential SARS-CoV-2 inhibitors [86]. In the last VS study, two promising compounds were identified for the treatment of Alzheimer’s disease [90].

The compounds of CIFPMA have been tested in over 25 in vitro and in vivo bioassays for different therapeutic targets, including anti-HIV (human immunodeficiency virus), antioxidants and anticancer [73]. 

In the UNIIQUIM database, molecules were found with potential analgesic activity [88].

In the BIOFACQUIM database, eight beta-glucosidase inhibitors were identified. The pharmacological applications of these compounds include obesity, diabetes, hyperlipoproteinemia, cancer, HIV and hepatitis B and C [87]. In another study, three compounds were identified to prevent and improve multiple adverse outcomes related to age [89]. The identified compounds from Latin American NP databases are in the Table 1.

### 2.2. Dataset Curation

**Figure 2:**
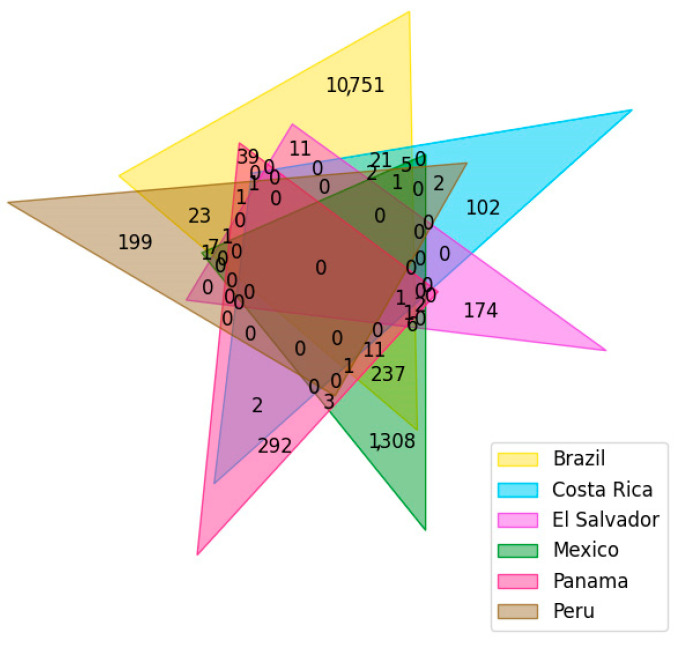
Venn diagram showing the number of unique and overlapping compounds contained in the nine Latin American databases.

From nine Latin American NP databases of six different countries (Table 2), the first version of LANaPDB, which currently contains 12959 compounds in total, was constructed. The number of unique and overlapping compounds is shown in the Figure 2. The number of unique compounds is proportionally similar to the number of compounds contained in the databases that comprise every country.

### 2.3. Structural Classification

**Figure 3:**
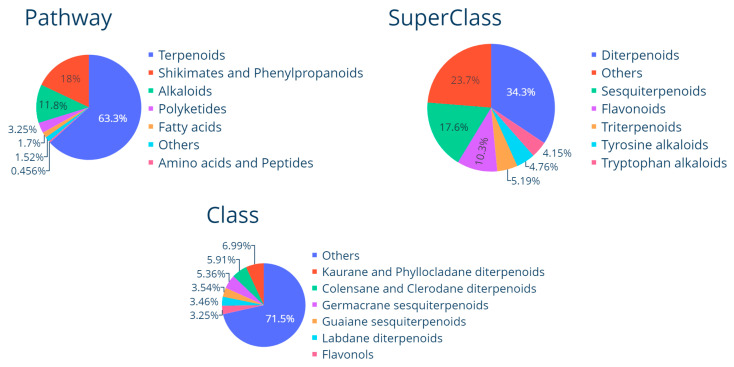
Structural classification of the compounds in the current (first) version of LANaPDB.

The compounds were classified in a total of seven different pathways, fifty-three superclasses and three hundred and thirty-six classes (Figure 3). The three predominant pathways are terpenoids (63.2%), shikimates and phenylpropanoids (18%) and alkaloids (11.8%). The main superclasses are diterpenoids (34.3%), sesquiterpenoids (17.6%) and flavonoids (10.3%). The prevalent classes are kaurane and phyllocladane diterpenoids (6.99%), colensane and clerodane diterpenoids (5.91%) and germacrane sesquiterpenoids (5.36%). The results are in accordance with expectations because the terpenoids are the most diverse group of secondary metabolites derived from natural sources [101].

### 2.4. Physicochemical Properties

**Figure 4:**
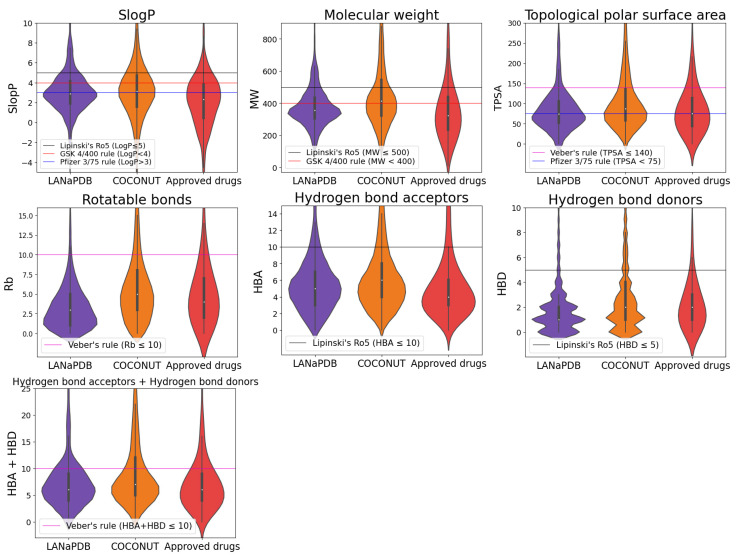
Violin plots summarizing the distribution of the representing physicochemical properties of pharmaceutical interest of the compounds of three databases LANaPDB, COCONUT and FDA-approved small molecule drugs.

**Figure 5:**
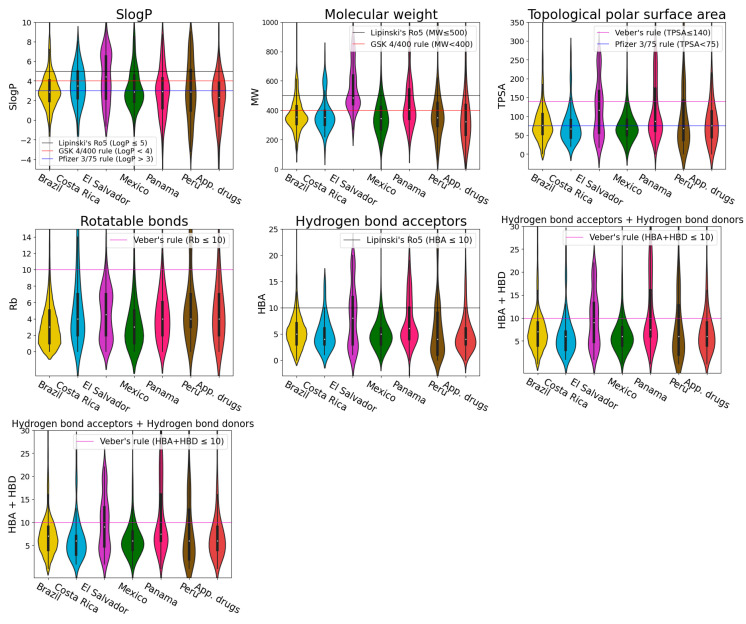
Violin plots summarizing the distribution of the physicochemical properties of pharmaceutical interest of the compounds in LANaPDB and FDA-approved small molecule drugs (App. drugs). The databases that encompass LANaPDB for every country: Brazil (NuBBE_DB_, SistematX and UEFS), Costa Rica (NAPRORE-CR), El Salvador (LAIPNUDELSAV), Mexico (UNIIQUIM and BIOFACQUIM), Panama (CIFPMA) and Peru (PeruNPDB).

The violin plots show the distribution of six physicochemical properties of pharmaceutical interest: SlogP [102], molecular weight (MW), topological polar surface area (TPSA) [103], rotatable bonds (Rb), hydrogen bond acceptors (HBA) and hydrogen bond donors (HBD) (Figure 4 and Figure 5). In the violin plots, the limits of the following rules of thumb of drug-likeness are marked with a horizontal line: Lipinski’s rule of 5 (Ro5) [104,105], Veber’s rules [106], GlaxoSmithKline’s (GSK) 4/400 rule [107] and Pfizer 3/75 rule [108] (Table S2). Physicochemical properties in the limits of either Lipinski’s, Veber’s or GSK rules is usually related with a good oral bioavailability. The fulfillment of these rules of thumb is associated with the improvement of the following parameters: aqueous solubility and intestinal permeability (Lipinski’s Ro5); passive membrane permeation (Veber’s rules); absorption, distribution, metabolism, excretion and toxicity (ADMET) profile (GlaxoSmithKline’s 4/400 rule); and toxicity (Pfizer 3/75 rule).

**Figure 6:**
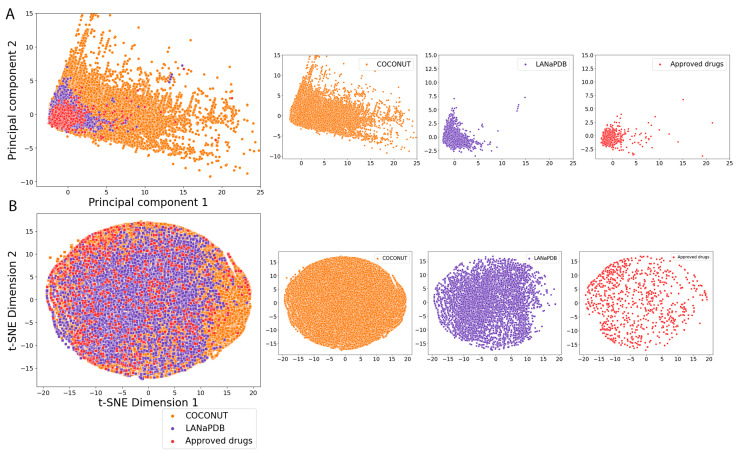
Visual representation of the chemical space based on six physicochemical properties of pharmaceutical interest of LANaPDB and its comparison with COCONUT and approved drugs. The chemical space was generated with (**A**) principal component analysis (PCA), the first two principal components capture 89.3% of the total variance; (**B**) t-distributed stochastic neighbor embedding (t-SNE).

NPs contain complex structures and are large and diverse; therefore, compared with synthetic drugs, it is not easy for them to satisfy most of the criteria of Lipinski’s Ro5 [109] or the other drug-likeness parameters mentioned above. Nevertheless, it is shown in the violin plots that a broad range of the LANaPDB compounds satisfy most of the rules of thumb of Table S2 for the physicochemical properties of pharmaceutical interest. The LANaPDB and COCONUT compound distributions of the physicochemical properties are, in general, similar (Figure 4). Additionally, as expected, COCONUT covers the broadest area of the chemical space, because it is the largest database (411,000 compounds) (Figure 6). Many compounds of LANaPDB fulfill the rules of thumb associated with drug-likeness (Figure 4) and part of the LANaPDB chemical space overlaps with the chemical space comprised by the approved drugs (Figure 6).

**Figure 7:**
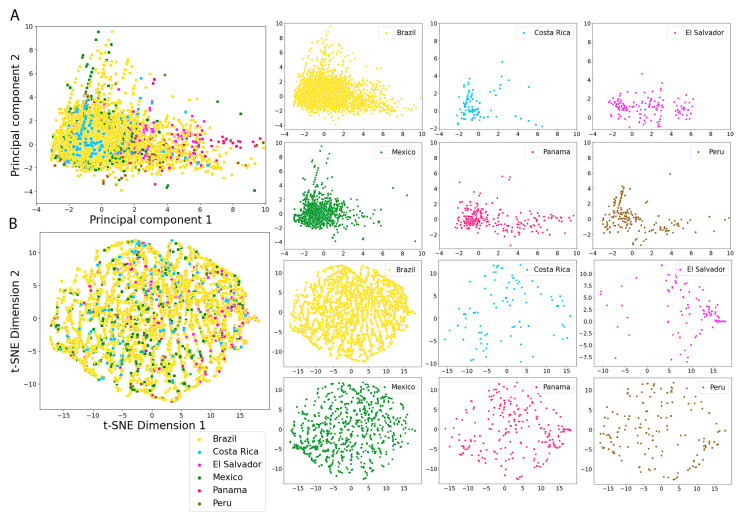
Visual representation of the chemical space based on six physicochemical properties of pharmaceutical interest of LANaPDB and individual Latin American natural product databases. The chemical space was generated with (**A**) principal component analysis (PCA), with the first two principal components capturing 84.6% of the total variance; (**B**) t-distributed stochastic neighbor embedding (t-SNE).

The distribution of the physicochemical properties of the NPs in the countries with more compounds (Brazil and Mexico) is, in general, more focused in certain regions, compared with the NPs from countries with less compounds (Costa Rica, El Salvador, Panama and Peru) which are more broadly distributed (e.g., SlogP, Brazil vs Peru from Figure 5). Panama and Peru show similar distributions in all the physicochemical properties, which may be due to the similar distribution of the dominant structural features in both datasets (Panama: 42% shikimates and phenylpropanoids and 34.3% terpenoids; Peru: 29.6% shikimates and phenylpropanoids and 39.2% terpenoids). In general, the distributions of physicochemical properties of the Latin American countries and the approved drugs are focused in the same regions (Figure 5). The chemical space represented by the six physicochemical properties is overlapped among the NPs from the six Latin American countries (Figure 7). In the principal component analysis (PCA), the first two principal components are enough to represent most of the explained variance percentage: 89.3% in the LANaPDB, COCONUT and approved drugs comparison (Figure 6A) and 84.6% in the Latin American countries comparison (Figure 7A). Moreover, TPSA, MW, HBD and HBA are the descriptors with greater contributions to principal component 1. The descriptors with greater contributions to principal component 2 are SlogP and Rb (Table S3).

### 2.5. Molecular Fingerprints

**Figure 8:**
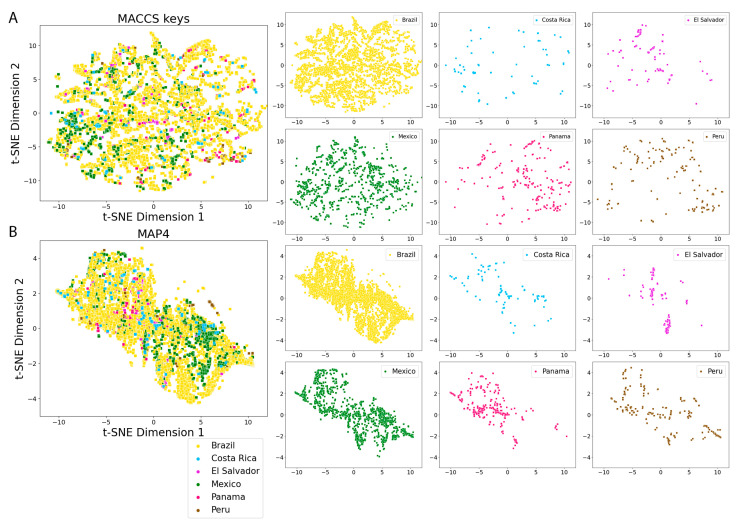
Visual representation of the chemical multiverse of LANaPDB and individual Latin American natural product databases. The chemical multiverse is a group of chemical spaces, each generated with a different set of descriptors. Chemical space comprised by (**A**) (t-SNE)-MACCS keys (166-bits) fingerprint; (**B**) (t-SNE)-MAP4 fingerprint.

**Figure 9:**
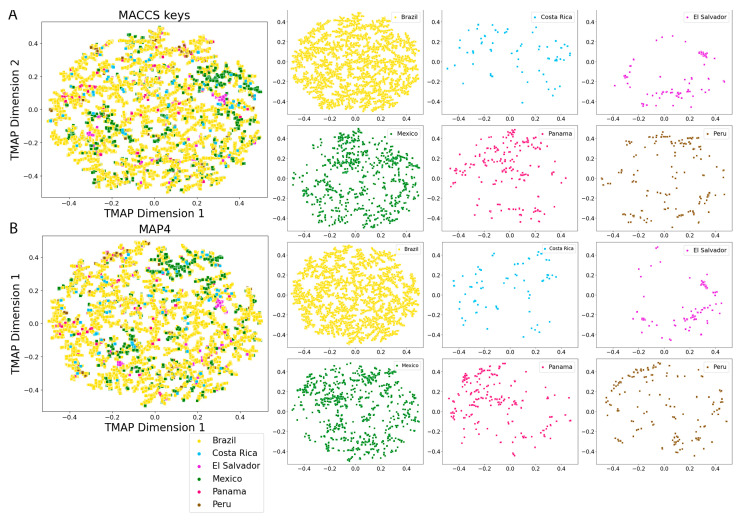
Visual representation of the chemical multiverse of LANaPDB and individual Latin American natural product databases. The chemical multiverse is a group of chemical spaces, each generated with a different set of descriptors. Chemical space comprised by (**A**) (TMAP)-MACCS keys (166-bits) fingerprint; (**B**) (TMAP)-MAP4 fingerprint (interactive version: https://github.com/alexgoga21/LaNaPDB/blob/main/Interactive%20TMAP_MAP4.html) (accessed on 28 September 2023).

Figure 8 and Figure 9 show the visual representation of the chemical multiverse of LANaPDB generated with t-distributed stochastic neighbor embedding (t-SNE) and tree MAP (TMAP) [110] and two fingerprints of different designs: MACCS keys (166-bits) (Figure 8A and Figure 9A) and MAP4 (Figure 8B and Figure 9B). As discussed recently, the chemical multiverse can be defined as a group of chemical spaces, each generated with a diverse set of descriptors [100]. A chemical multiverse is a natural extension of the concept of chemical space and its advantage is that it provides a more complete description of the chemical space of a set of compounds as opposed to using only one representation. Moreover, substructure fingerprints perform best for small molecules, such as drugs, while atom–pair fingerprints are preferable for large molecules. Given that it is common to see large molecules among the NPs, MAP4 fingerprint was chosen because it is suitable for both small and large molecules by combining substructure and atom–pair concepts [111]. The MACCS keys (166-bits) [112] fingerprint was employed to compare the results obtained with the MAP4 fingerprint with a well-known substructure fingerprint. 

**Figure 10:**
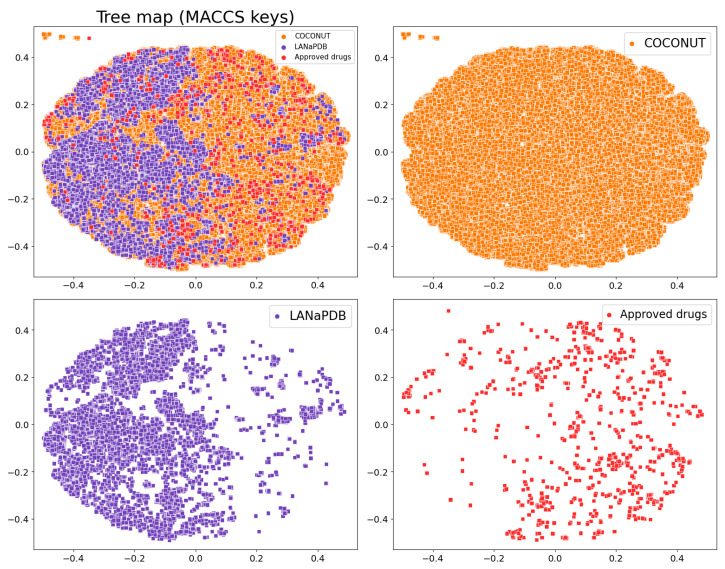
Tree map from MACCS keys (166-bits) of LANaPDB and the comparison with COCONUT and approved drugs.

Based on the visual representation of the chemical multiverse, it is concluded that t-SNE has a better performance with MACCS keys (166-bits) fingerprint over MAP4 fingerprint, separating the NPs on clusters according to the structural features (Figure 8). The efficacy of TMAP to separate compounds in clusters from MACCS keys (166-bits) and MAP4 fingerprints is similar with both fingerprints (Figure 9). Moreover, TMAP performed better than t-SNE in the NPs cluster creation with both fingerprints. An interactive version of the scatter plot created with TMAP from MAP4 fingerprints (Figure 9B) is freely available at https://github.com/alexgoga21/LaNaPDB/blob/main/Interactive%20TMAP_MAP4.html (accessed on 28 September 2023). To open the interactive map, download the file and open it in a web explorer. Given that TMAP performed better than t-SNE, and MACCS keys (166-bits) and MAP4 fingerprints showed a similar efficacy in the TMAP, the comparison of LANaPDB with the reference databases was made with TMAP and MACCS keys (166-bits) fingerprint (Figure 10). It can be observed that LANaPDB overlaps with COCONUT in well-defined areas; nevertheless, the approved drugs are more dispersed and some of them overlap with the compounds in LANaPDB (Figure 10).

## 3. Materials and Methods

The visual representations of the different chemical spaces that consider either physicochemical properties or molecular fingerprints are illustrated with scatter plots (Figure 6, Figure 7, Figure 8, Figure 9 and Figure 10). Every point in the scatter plots represents a unique compound. The scatter plots were created in the python programming language (version 3.10.7), employing the seaborn module (0.12.2) [113].

### 3.1. Dataset Curation

The Latin American NP databases of Table 2 were used to construct the unified NP database LANaPDB. The process was carried out in the python programming language (version 3.10.7), employing the RDKit (version 2022.03.5) [114] and MolVS (version 0.1.1) [115] modules. The standardization process of MolVS was applied, which consisted of the remotion of explicit hydrogens, disconnection of covalent bonds between metals and organic atoms (the disconnected metal is not preserved), application of normalization rules (transformations to correct common drawing errors and standardization of functional groups), reionization (ensure the strongest acid groups protonate first in partially ionized molecules) and recalculation of the stereochemistry (ensures preservation of the original stereochemistry). The salts were removed, keeping the largest fragment, which was neutralized, and the remaining partially ionized fragments were reionized. The canonical tautomer was determined, and, from the InChIKey strings of the canonical tautomer, the duplicate compounds were removed. 

A Venn diagram was constructed with python programming language, employing the Venn module (version 0.1.3), to see the number of unique and overlapping compounds along the nine Latin American databases.

### 3.2. Structural Classification

Compounds in LANaPDB were classified with NPClassifier [116], which is a freely available deep neural network-based structural classification tool for NPs. NPClassifier establishes a classification system based on the literature from the specialized metabolism of plants, marine organisms, fungi and microorganisms. The categories used in NPClassifier were defined at three hierarchical levels: pathway (nature of the biosynthetic pathway), superclass (chemical properties or chemotaxonomic information) and class (structural details). Pie charts were constructed with the python programming language (version 3.10.7), employing the Plotly express module (version 0.4.1) [117], to represent the distribution of the dominant structures for each of the three categories.

### 3.3. Physicochemical Properties

Employing the software KNIME [118] version 4.7.1, with the RDKit nodes, six physicochemical properties of pharmaceutical interest were calculated: SlogP [102], MW, TPSA [103], Rb, HBA, HBD. Violin plots were constructed to summarize the distribution of each property individually. In each violin plot, we highlighted the limit of drug-like rules of thumb (Table S2). To generate a visual representation of the chemical space of the compound libraries based on the six properties, we reduced the data dimensionality to two dimensions, employing PCA and t-SNE with the python module Scikit-learn version 1.2.2 [119]. PCA: principal component one and principal component two to represent the six physicochemical properties. t-SNE hyperparameters: perplexity = 40 and number of iterations = 300. The distribution of the individual properties and the two-dimensional representation of the chemical space were conducted to analyze and compare the properties of the NPs among the six Latin American countries and with two other reference datasets, COCONUT [40] and FDA-approved small-molecule drugs, version 5.1.10 (released by DrugBank in January 2023) [120].

### 3.4. Molecular Fingerprints

A fingerprint encodes the structural information of a molecule in a vector [121]. Two different fingerprints were determined for each molecule: MACCS keys (166-bits) fingerprint and MAP4 fingerprint. MACCS keys (166-bits) fingerprints were calculated with KNIME [118] version 4.7.1, employing the Chemistry Development Kit (CDK) nodes [122]. MAP4 fingerprints were determined with the python programming language, following the instructions of the creators of this fingerprint [111]. To allow a 2D representation of the molecules, two different techniques for dimensionality reduction were employed: t-SNE and TMAP [110]. For t-SNE, the same hyperparameters of Section 3.3. were used. Employing the TMAP with MACCS keys (166-bits), LANaPDB was compared with the two reference datasets used in Section 3.3. From the MAP4 fingerprints, an interactive TMAP was created in the python programming language (version 3.9.17) with the faerun module (version 0.4.2).

## 4. Conclusions

Here we report progress towards the assembly of the first version of a unified Latin American Natural Products Database. The current version has 12,959 compounds from nine compound databases of six different Latin American countries. The database is freely available and the information of each compound in this first version includes the structures in SMILES format, the structural classification and six physicochemical properties of pharmaceutical interest. The LANaPDB compounds are produced by plants, terrestrial and marine animals, fungi and bacteria. Moreover, the most abundant NPs were the terpenoids (63.2%), followed by the shikimates and phenylpropanoids (18%) and the alkaloids (11.8%). Although it is not easy for NPs to fulfill most of the drug-likeness parameters compared with synthetic drugs, many LANaPDB compounds satisfy some drug-like rules of thumb for physicochemical properties. Moreover, the chemical space covered by LANaPDB completely overlaps with COCONUT and, in some regions, with the FDA-approved drugs. The concept of the chemical multiverse was used to generate multiple chemical spaces from two different dimensionality reduction techniques (t-SNE and TMAP) and two fingerprints (MACCS keys (166-bits) and MAP4). MAP4 performed better than t-SNE in separating the compounds into clusters according to their structural features. All the resources used for the assembly, curation, analysis and graphics creation are freely available. 

LANaPDB is part of one of the strategic actions to contribute to the further development of chemoinformatics and related disciplines in Latin America and strengthen the interactions between Latin America and other geographical regions [123]. We encourage the community to visit the websites where the individual NP databases of the different Latin American countries are available (Table S1).

We anticipate that LANaPDB will continue growing and evolving with the update of more compounds from each existing database, along with the addition of databases from other Latin American countries. One of the first steps in this direction is the integration of a larger set of NAPRORE-CR and the incorporation of natural products database NPDB-EjeCol from Colombia. Another perspective is the implementation of the database in a free web server. Likewise, LANaPDB could be integrated with other large public databases of natural products, such as COCONUT or LOTUS.

## Figures and Tables

**Table 1:** Bioactive compounds found from the Latin American Natural Products Databases.

Database Name	Disease or Symptom	Number of Identified Compounds	Reference
NuBBE_DB_	Chagas disease	10	[80]
	Tuberculosis	13	[82]
SistematX	Chagas disease	13	[81]
	Leishmaniasis	13	[83]
	Schistosomiasis	5	[85]
	Coronavirus disease 2019	19	[86]
	Alzheimer’s disease	2	[90]
UNIIQUIM	Pain	6	[88]
BIOFACQUIM	Obesity	8	[87]
	Diabetes		
	Hyperlipoproteinemia Cancer		
	HIV/AIDS *		
	Hepatitis B and C.		
	Age-related diseases	3	[89]

* Human immunodeficiency virus infection and acquired immunodeficiency syndrome (HIV/AIDS).

**Table 2:** Latin American Natural Product Databases analyzed in this work.

Database Name (Country)	Number of Compounds ^a^	Source	General Description	References
NuBBE_DB_ (Brazil)	2223	Plants Microorganisms Terrestrial and marine animals	Natural products of Brazilian biodiversity. Developed by the São Paulo State University and the University of São Paulo.	[68,69]
SistematX (Brazil)	9514	Plants	Database composed of secondary metabolites and developed at the Federal University of Paraiba.	[70,71]
UEFS (Brazil)	503	Plants	Natural products that have been separately published, but there is no common publication nor public database for it. Developed at the State University of Feira de Santana.	[72]
NAPRORE-CR (Costa Rica)	359	Plants Microorganisms	Developed in the CBio3 and LaToxCIA Laboratories of the University of Costa Rica.	*
LAIPNUDELSAV (El Salvador)	214	Plants	Developed by the Research Laboratory in Natural Products of the University of El Salvador.	*
UNIIQUIM (Mexico)	1112	Plants	Natural products isolated and characterized at the Institute of Chemistry of the National Autonomous University of Mexico.	[76]
BIOFACQUIM (Mexico)	553	Plants Fungus Propolis Marine animals	Natural products isolated and characterized in Mexico at the School of Chemistry of the National Autonomous University of Mexico and other Mexican institutions.	[77,78]
CIFPMA (Panama)	363	Plants	Natural products that have been tested in over twenty-five in vitro and in vivo bioassays for different therapeutic targets. Developed at the University of Panama.	[73,74]
PeruNPDB (Peru)	280	Animals Plants	Created and curated at the Catholic University of Santa Maria.	[75]

The URL of the websites where the natural product databases of Latin America are allocated is in the Supplementary Material (Table S1). ^a^ Number of compounds contained in each database previous to the curation process. * Actually, there is not a publication associated with the database.

## Data Availability

Data are contained within the article, Supplementary Material and the following github repository https://github.com/alexgoga21/LaNaPDB (accessed on 28 September 2023).

## Supplementary Materials

The following supporting information can be downloaded at: https://www.mdpi.com/article/10.3390/ph16101388/s1, Table S1: Websites of the natural product databases of Latin America; Table S2: Rules of thumb guides associated with drug-likeness; Table S3: Analysis metrics of the principal component analysis.
