# Supplementary material for: Navigating the Chemical Space and Chemical Multiverse of a Unified Latin American Natural Product Database: LANaPDB
Source: Pharmaceuticals (Basel). 2023 Sep 30;16(10):1388. doi: 10.3390/ph16101388 (PMC10609821; doi:10.3390/ph16101388)
Supplement: Supplementary file 1 [file pharmaceuticals-16-01388-s001.zip › pharmaceuticals-2603413-supplementary.pdf]

SUPPLEMENTARY MATERIAL

Table S1. Websites of the natural product databases of Latin America.

**Database name**	**Country**	**Database website**
NaturAr	Argentina	https://naturar.quimica.unlp.edu.ar/es/
NuBBE$_{DB}$	Brazil	http://nubbe.iq.unesp.br/portal/nubbe-search.html
SistematX	Brazil	https://sistematx.ufpb.br/
UEFS	Brazil	http://zinc12.docking.org/catalogs/uefsnp
NAPRORE-CR	Costa Rica	*
LAIPNUDELSAV	El Salvador	*
UNIIQUIM	Mexico	https://uniiquim.iquimica.unam.mx/
BIOFACQUIM	Mexico	Database version 2 https://figshare.com/articles/dataset/BIOFACQUIM_V2_sdf/11312702
CIFPMA	Panama	*
PeruNPDB	Peru	https://perunpdb.com.pe/

*No available.

Table S2. Rules of thumb - guides - associated with drug-likeness.

	Molecular weight	LogP	Number of hydrogen bond acceptors	Number of hydrogen bond donors	Sum of hydrogen bond acceptors and donors	Topological polar surface area	Number of rotatable bonds	References
Lipinski's rule of 5	≤ 500	≤ 5	≤ 10	≤ 5				[1,2]
Veber's rules					≤ 12	≤ 140	≤ 10	[3]
GlaxoSmithKline's 4/400 rule	<400	<4						[4]
Pfizer 3/75 rule		>3				< 75		[5]

Table S3. Analysis metrics of the principal component analysis.

Descriptors	LaNaPDB, COCONUT and approved drugs comparison		Latin American countries comparison	
	PC1*	PC2**	PC1	PC2
SlogP	0.14028102	-0.79233759	0.23778911	-0.67762065
Topological polar surface area	-0.4891108	0.11257005	-0.51947212	0.08670239
Molecular weight	-0.45033318	-0.27365197	-0.42878314	-0.37683651
Number of rotatable bonds	-0.31703665	-0.50279962	-0.24992425	-0.58680117
Number of hydrogen bond donors	-0.4633437	0.14429034	-0.41631399	0.21532234
Number of hydrogen bond acceptors	-0.47232644	0.10495983	-0.50395496	0.02465498
Explained variance percentage	67.1%	22.2%	59.8%	24.8%
Sum of PC1 and PC2 explained variance percentage	89.3%		84.6%	

*Principal component 1; **Principal component 2

References

1. Lipinski, C.A.; Lombardo, F.; Dominy, B.W.; Feeney, P.J. Experimental and computational approaches to estimate solubility and permeability in drug discovery and development settings. *Adv. Drug Deliv. Rev.* **2001**, *46*, 3–26, doi:10.1016/S0169-409X(00)00129-0.

2. Lipinski, C.A. Lead- and drug-like compounds: the rule-of-five revolution. *Drug Discov. Today Technol.* **2004**, *1*, 337–341, doi:10.1016/j.ddtec.2004.11.007.

3. Veber, D.F.; Johnson, S.R.; Cheng, H.-Y.; Smith, B.R.; Ward, K.W.; Kopple, K.D. Molecular properties that influence the oral bioavailability of drug candidates. *J. Med. Chem.* **2002**, *45*, 2615–2623, doi:10.1021/jm020017n.

4. Gleeson, M.P. Generation of a set of simple, interpretable ADMET rules of thumb. *J. Med. Chem.* **2008**, *51*, 817–834, doi:10.1021/jm701122q.

5. Hughes, J.D.; Blagg, J.; Price, D.A.; Bailey, S.; Decrescenzo, G.A.; Devraj, R.V.; Ellsworth, E.; Fobian, Y.M.; Gibbs, M.E.; Gilles, R.W.; Greene, N.; Huang, E.; Krieger-Burke, T.; Loesel, J.; Wager, T.; Whiteley, L.; Zhang, Y. Physiochemical drug properties associated with in vivo toxicological outcomes. *Bioorg. Med. Chem. Lett.* **2008**, *18*, 4872–4875, doi:10.1016/j.bmcl.2008.07.071.
